# Effects of litter feeders on the transfer of ^137^Cs to plants

**DOI:** 10.1038/s41598-018-25105-4

**Published:** 2018-04-27

**Authors:** Nobuyoshi Ishii, Masashi Murakami, Takahiro Suzuki, Keiko Tagami, Shigeo Uchida, Nobuhito Ohte

**Affiliations:** 1 National Institute of Radiological Sciences, 4-9-1 Anagawa, Inage-ku, Chiba, 263-8555 Japan; 2 Chiba University, 1-33 Yayoi-cho, Inage-ku, Chiba, 263-8522 Japan; 3 The University of Tokyo, 1-1-1, Yayoi, Bunkyo-ku, Tokyo, 113-8657 Japan

## Abstract

The effects of the Japanese horned beetle larvae on the transfer of ^137^Cs from a contaminated leaf litter to the leaf vegetable, komatsuna (*Brassica rapa var. perviridis*) was studied. Feces of the larvae which were fed ^137^Cs-contaminated leaf litter were added to a potting mix in which komatsuna plants were cultivated. The presence of feces increased the harvest yield of komatsuna, suggesting that feces provided nutrients for the plant growth. In addition, the amount of exchangeable ^137^Cs in leaf litter was experimentally confirmed to be enhanced by the presence of feces which were excreted by larvae feeding. However, there was no difference in the soil-to-plant transfer factor of ^137^Cs for the presence and absence of feces. Interactions between clay minerals and exchangeable ^137^Cs in the soil beneath the litter layer may diminish the root uptake of ^137^Cs. From these results, it was concluded that the effect of exchangeable ^137^Cs released from feces was limited for the transfer of ^137^Cs to plants if plant roots were not present in litter layers.

## Introduction

Large amounts of radionuclides were released to the environment by the accident at TEPCO’s Fukushima Daiichi Nuclear Power Plant (FDNPP) after the Tohoku earthquake on 11 March 2011. The Nuclear and Industrial Safety Agency of Japan (NISA) estimated that the total released amounts from the FDNPP were approximately 160 PBq for ^131^I and 15 PBq for ^137^Cs^1^. Large areas surrounding the FDNPP and areas in neighboring prefectures were contaminated with fallout radionuclides^2^. The central and local governments have been making effort to lower exposure to radiation in living areas and agricultural fields^3,4^. For example, the removal of topsoil, abrasive brush sweeping of asphalt-paved roads, and the use of high-pressure water cleaners on roofs and other surfaces have been performed. In contrast to living areas, there has been little decontamination of forests. Hashimoto *et al*.^5^ estimated that 428 km^2^ of the forests in Fukushima had been contaminated with ≥1,000 kBq m^−2^ of radiocesium (^134^Cs and ^137^Cs).

Fukushima Prefecture has a large forest area (9,361 km^2^ in 2010), and forestry is one of its major industries. In addition, forests supply products such as mushrooms, wild plant shoots, and game animals. The contamination of these wild foods by radiocesium is a major public concern because they present a possible route for radiocesium to enter the human food chain. To reduce this concern, and to ensure better decontamination measures, it is important to understand the behavior of ^137^Cs in forest ecosystems. After the FDNPP accident, fallout ^137^Cs was mainly intercepted by forest canopies^6^. Some of the contaminated leaves have since fallen from the trees, and have formed leaf litter with decomposed humus on forest floors which currently constitute the main sink for ^137^Cs^7^. Therefore, the mobility of the ^137^Cs in leaf litter is one of the key factors governing the transfer of ^137^Cs to other forest components.

The Japanese horned beetle, *Trypoxylus dichotomus*, widely inhabits forests in Japan. Their larvae are saprophagous and thus feed on and decompose decaying plant materials and leaf litters. The main function of decomposition processes in forest ecosystems is the release of nutrients from litters^8^, and some of these nutrients, including radiocesium, will be taken up by plants and trees. Surely, the effect of feces of saprophage organisms on the transfer of ^137^Cs to growing plants following its release is poorly understood.

The aim of the present study was to demonstrate whether larval feces resulting from decomposition of contaminated leaf litters can promote the release of ^137^Cs from leaf litter and subsequent transfer of the released ^137^Cs to plants. The leaf vegetable, komatsuna (*Brassica rapa var. perviridis*) was cultivated in potting mixes with and without feces of beetle larvae. After harvesting the komatsuna, the concentrations of ^137^Cs in plants and potting mixes were measured, and soil-plant transfer factors were determined.

## Results and Discussion

### Decomposition of leaf litter

Larvae were maintained on the ^137^Cs-intact-litter bed (radiocesium contaminated uncrushable litter bed) to obtain decomposed leaf litter. No larvae mortality was observed, and they did not reach the prepupal state during the rearing period. Culture conditions, therefore, were suitable for larvae survival.

**Figure 1:**
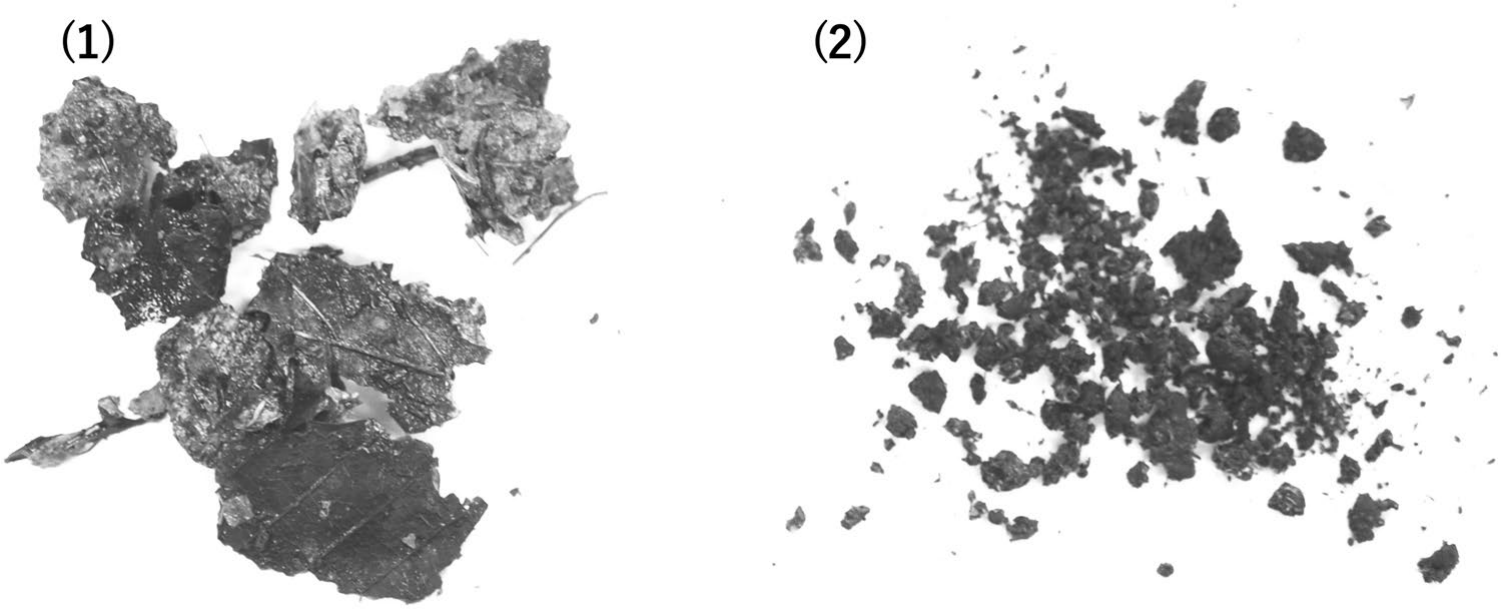
Photos of (**1**) intact leaf litter and (**2**) decomposed litter.

The ^137^Cs-intact-litter was physically broken down into small pieces by larval feeding (Fig. 1). Some of the litter was chemically decomposed through digestive processes and then excreted as feces. Feces characteristically had a black columnar shape, and were present in the rearing vessels at the end of rearing period. Therefore, the decomposed-litter-mixture contained physically and chemically decomposed leaf litter.

### Harvest yield

**Figure 2:**
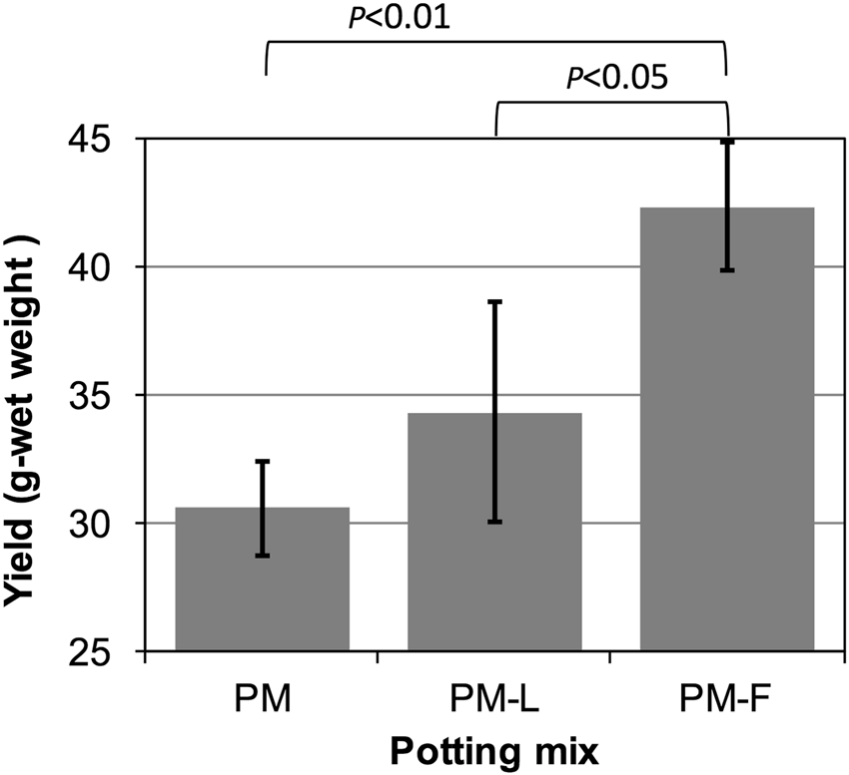
The amounts of harvested komatsuna from the PM (a base potting mix), PM-L (PM with the ^137^Cs-intact-litter) and PM-F (PM with the decomposed-litter-mixture) potting mixes. The fresh weights of komatsuna grown in PM, PM-L, and PM-F were 30.6 ± 1.8 g, 34.3 ± 4.3 g, and 42.3 ± 2.5 g, respectively. Error bars show standard deviation (n = 3).

Komatsuna was successfully grown in the three types of potting mixes, but the yield at harvest differed among them (Fig. 2). ANOVA of the data confirmed that the amount of komatsuna grown in the PM-F soil was significantly larger than that in the other two mixes (*p* < 0.05 for vs. PM-L and *p* < 0.01 for vs. PM). No difference in the yield was found between komatsuna plants grown in PM and on PM-L. Because PM-L is a blended soil including PM and the ^137^Cs-intact-litter, the results implied that the addition of 44 g of the ^137^Cs-intact-litter had no effect on the amount of yield under the present experimental conditions. On the other hand, the decomposed-litter-mixture increased the harvest yield, and that suggested the decomposed-litter-mixture had plant growth-promoting abilities. The increase in the yield could be caused by the release of nutrients resulting from the decomposition of the ^137^Cs-intact-litter by the beetle larvae; such release of nutrients like nitrogen, phosphorus, and potassium from leaf litter through decomposition has been demonstrated^8,9^. It was also possible that release of ^137^Cs occurred, as leaching of ^137^Cs from leaf litter after being submerged in water has been reported^10,11^.

### Extraction of ^137^Cs from the ^137^Cs-intact-litter and the decomposed-litter-mixture samples

**Figure 3:**
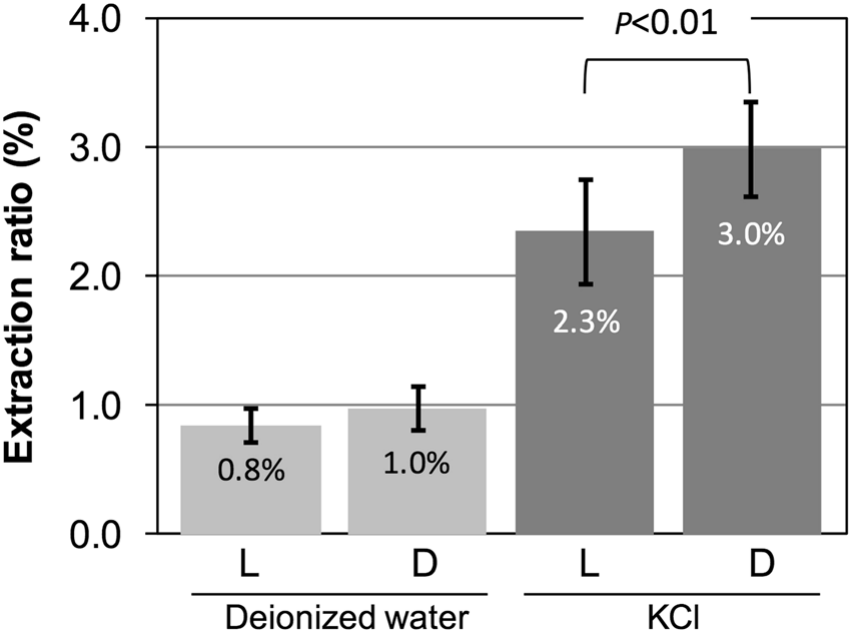
Extraction ratios of ^137^Cs from (L) the ^137^Cs-intact-litter and (D) the decomposed-litter-mixture. Deionized water and 2 M KCl were separately used as eluents. Error bars show standard deviation (n = 9). Significant increase in the extraction ratio was observed for the decomposed litter mixture which was extracted by the KCl solution.

An extraction experiment was conducted to confirm the effect of biological decomposition on the release of ^137^Cs from the ^137^Cs-intact-litter samples. In the experiment, eluents (deionized water and KCl solution) were passed through a syringe containing the ^137^Cs-intact-litter or the decomposed-litter-mixture, so there was a contact-time between eluents and both litter samples of several minutes. Despite such a short contact-time, ^137^Cs was detected in the dissolved fraction for all samples tested (Fig. 3). Sakai *et al*.^11^ showed that the amount of extractable ^137^Cs from litter increased with soaking time. The amount of released ^137^Cs from ^137^Cs-intact-litter may be enhanced under natural conditions, because the litter layer in the natural environment generally holds moisture allowing a prolonged contact-time of the ^137^Cs-intact-litter with water. The contaminated leaf litter, therefore, becomes a potential source of ^137^Cs for plants even if that litter is not biologically decomposed.

Availability of radiocesium for plant is affected by the chemical form of the radiocesium. Although the physical size of ^137^Cs in the dissolved fraction was less than 0.45 µm, its chemical form was not evaluated. Tagami and Uchida^12^ demonstrated that radiocesium in the <0.45 µm fraction was mostly in the Cs^+^ form. In their study ^137^Cs was extracted from leaf litter by deionized water and filtered through a membrane filter with 0.45-µm-pores. This treatment procedure was similar to that of the present experiment, so the extracted ^137^Cs in the study was probably in a cationic form which is available for uptake by plants.

The effect of biological decomposition on the extraction of ^137^Cs was also confirmed. Decomposition of the ^137^Cs-intact-litter by larvae significantly increased the extraction ratio of ^137^Cs from 2.3 ± 0.4% to 3.0 ± 0.4% in the exchangeable fraction by KCl (Fig. 3). On the other hand, no difference was found for the extraction ratios of the soluble fraction (extracted with deionized water). In comparison to deionized water, the effect of KCl as extractant was evident. For example, extraction ratios by KCl for the ^137^Cs-intact-litter samples were 2.8 times those by deionized water. Similarly, when ^137^Cs was extracted from the decomposed-litter-mixture samples by KCl, 3.1 times higher amounts of ^137^Cs were extracted in comparison with those extracted by deionized water. In addition, the difference in these extraction efficiencies (the ratio of ^137^Cs extracted by KCl to ^137^Cs extracted by deionized water) of 2.8 and 3.1 suggested that decomposition of the ^137^Cs-intact-litter by larvae promoted the production of the exchangeable ^137^Cs. Because simple physical decomposition affects chemical composition of the ^137^Cs-intact-litter, digestive processes of larvae may cause a change in the chemical composition of the ^137^Cs-intact-litter. Consequently, the amount of ^137^Cs in the exchangeable fraction increased for the decomposed-litter-mixture samples. Some previous studies also observed changes in chemical composition of soil^13^ and litter^14,15^ by digestion. Additionally, Coeurdassier *et al*.^16^ showed an increase of the water-soluble cadmium fraction in soil with earthworms. These examples suggest that digestive processes commonly play an important role in the change of chemical composition.

### Effect of beetle larvae on ^137^Cs transfer from soil to plant

An increase in the amount of exchangeable ^137^Cs may enhance the transfer of ^137^Cs to plants. An increase in the ^137^Cs concentration in rice plants was observed when increasing the amount of exchangeable ^137^Cs in soil^17^. To confirm the effect of feeding by beetle larvae on the transfer of ^137^Cs, komatsuna plants were grown in the PM-L and PM-F potting mixes. The activity concentrations of ^137^Cs in these potting mixes and plants on the day of harvest are shown in Table 1. The ^137^Cs activity concentrations for the both potting mixes were similar. There were no differences in ^137^Cs activity concentrations in komatsuna plants grown in both PM-L and PM-F. These results suggested that the ^137^Cs-intact-litter was a source of ^137^Cs regardless of decomposition.

**Table 1:** Activity concentrations of ^137^Cs in the potting mixes and komatsuna plants, and transfer factors on the day of harvest.

Potting mix	Treatment	Activity concentration of ^137^Cs (mean ± sd Bq/kg-dry)		TF (mean ± sd)
		Soil	Plant	
PM-L	+Litter	2.2 × 10^3^ ± 1.4 × 10^2^	5.6 × 10^1^ ± 6.6 × 10^1^	2.5 × 10^−2^ ± 2.9 × 10^−3^
PM-F	+decomposed litter and feces	2.6 × 10^3^ ± 1.6 × 10^2^	6.8 × 10^1^ ± 6.2 × 10^1^	2.6 × 10^−2^ ± 2.0 × 10^−3^

TFs were determined using the activity concentrations of ^137^Cs in potting mixes and plants (Table 1). A TF value of 2.6 × 10^−2^ for the PM-F potting mix was similar to that for the PM-L potting mix (*t*-test, *P* > 0.05) in spite of the significant release of exchangeable ^137^Cs from the decomposed-litter-mixture. This was in agreement with a previous result showing no increase in TF of ^137^Cs from soil to lettuce even if saprophagous organisms (earthworms) were present in the soil^18^. The lack of difference in TF may be explained in two ways by the interaction between exchangeable ^137^Cs and soil minerals. Firstly, the feeding activity by beetle larvae may have enhanced the release of the exchangeable ^137^Cs from the ^137^Cs-intact-litter (Fig. 3). Some of the exchangeable ^137^Cs would be fixed by soil minerals immediately after its release, because ^137^Cs is easily sorbed on the surface of soil minerals by ion exchange and chemical sorption^19^, and held in frayed edge sites^20^. These sorption and fixation processes could decrease the bioavailability of ^137^Cs to plants. Secondly, the significant increase of exchangeable ^137^Cs caused by the larvae in the present study was too small to significantly affect the transfer of ^137^Cs.

The TFs of up to 0.026 obtained in this study were close to the minimum values reported in previous studies for plants belonging to the family Brassicaceae^21,22^. The difference in the contact area between the ^137^Cs source and plant roots may affect TF values. The previous studies used sand, loam, clay, and organic soils as the ^137^Cs source. In such cases, most of the plant roots were probably in contact with the ^137^Cs source. In the present study, 44 g of the decomposed-litter-mixture was mixed with 2.6 kg of soil. In this case, the volume of the decomposed-litter-mixture was small in comparison with that of the soil, and thus the contact area between plant roots and the ^137^Cs source was also small. If the ^137^Cs released from the decomposed-litter-mixture is fixed by the soil before the uptake of ^137^Cs by the roots, TF value becomes low. The positional relationship between plant roots and the ^137^Cs source is one of the factors controlling TF value. In the natural environment, plant and tree roots grow in humus and soil mineral layers beneath the litter layer^23^, so there is little direct contact between roots and a litter layer covering the soil.

## Conclusions

The present study showed that biological decomposition of the ^137^Cs-intact-litter by saprophagous beetle larvae enhanced the release of exchangeable ^137^Cs which is available for uptake by plants. Involvement of the larval digestive processes in the change in the chemical composition was suggested. Although the amount of ^137^Cs in the exchangeable fraction increased, no detectable effect on the transfer of ^137^Cs to plants was observed under the experimental conditions. Therefore, the effect of litter feeders on the transfer of ^137^Cs to plants was limited. In the present study, the decomposed-litter-mixture was blended with some soils, and the exchangeable ^137^Cs was probably fixed by clay minerals. Therefore, further studies on interactions between soil minerals and exchangeable ^137^Cs and the positional relationship between plant roots and litter will be important to understand the behavior of ^137^Cs in actual forest ecosystems.

## Methods

### Sampling locations

Leaf litter which was contaminated with radiocesium (hereafter ^137^Cs-intact-litter) was collected in a deciduous broadleaf forest (mainly *Quercus serrata*) of the Kami-Oguni river catchment (37°7′N, 140°6′E) in Fukushima Prefecture on 29 June 2012. This forest was located about 53 km north-west of the FDNPP. The concentration of ^137^Cs was 3.2 × 10^5^ Bq/kg-dry on the collection day.

Last instar larvae of Japanese horned beetle were collected in a mixed deciduous forest of Showa-no-Mori Park (35°31′N, 140°16′E), Chiba City, Japan in March 2013. The Ministry of Education, Culture, Sports, Science and Technology (MEXT) has reported^24^ that the area surrounding this park was contaminated with radiocesium (^137^Cs and ^134^Cs) at less than 10 × 10^4^ Bq/m^2^.

### Rearing of larvae

Each larva was grown on 50 g-wet weight of ^137^Cs-intact-litter in a polyethylene rearing vessel (500 cm^3^) at 25 °C under light (16 hours)-dark (8 hours) conditions for 14 days; nine replicates were prepared. To retain the moisture conditions, deionized water was sprayed into the rearing vessels every 2 days during the cultivation period. All feces pellets excreted by the larva were removed at day 9 because these feces would have been affected by the mater ingested before the controlled rearing was started. The feces pellets present in the vessel at the end of the rearing were, therefore, excreted after day 9. Finally, after removing the larvae, the remaining ^137^Cs-intact-litter, physically crushed litter, and feces pellets in the rearing vessel were well mixed (hereafter called the decomposed-litter-mixture). The decomposed-litter-mixture was used in the two types of experiments: extraction and planting. The ^137^Cs-intact-litter alone was also prepared as a negative control culture with nine replicates.

### Extraction experiment

Five grams-dry weight of the ^137^Cs-intact-litter or the decomposed-litter-mixture were put into a syringe (volume of 50 mL). The concentration of ^137^Cs was 3.2 × 10^2^ Bq/g-dry for both litter samples. The bottom of the syringe was filled with quartz wool to keep the pieces of the litter samples in the syringe. Eluent (100 mL) was poured into the syringe to extract ^137^Cs from the ^37^Cs-intact-litter and the decomposed-litter-mixture samples. Two kinds of eluents were used: deionized water and 2 M KCl solution. Deionized water and KCl were used to obtain the water soluble ^137^Cs and the exchangeable ^137^Cs, respectively. The solution passing through the quartz wool was further filtered through a membrane filter having 0.45-µm-pores. The plant uptake is presumably limited to soluble chemical species. Even particles/colloids smaller than 0.45-µm may not be taken up by plant roots. The final filtrate samples were collected in U8 type polystyrene containers for the measurement of the activity concentrations of ^137^Cs. The ratio of extraction was calculated as follows:

1 $${\rm{Extraction}}\,{\rm{ratio}}\,( \% )={C}_{e}/{C}_{0}\times 100$$

where *C*_*e*_ is the total amount of ^137^Cs in the dissolved fraction (<0.45 µm), and *C*_0_ is the total amount of ^137^Cs in 5 g of the ^137^Cs-intact-litter or the decomposed-litter-mixture. These extraction experiment was repeated nine times.

### Planting experiment

Leaf vegetable plants of the family Brassicaceae, komatsuna (*Brassica rapa* var*. perviridis*) were grown in three potting mixes: (1) a base potting mix (PM), (2) PM with the ^137^Cs-intact-litter (PM-L) and (3) PM with the decomposed-litter-mixture (PM-F). The PM consisted of Akadama soil, leaf mold, Kuro-tsuchi, and a chemical fertilizer (8% nitrogen, 8% phosphorus, and 8% potassium by weight). First, the Akadama soil, leaf mold, and Kuro-tsuchi, were mixed with a volume ratio of 6:3:1, and then 10 g of the chemical fertilizer was added to 2.6 kg of the soil mixture. For the preparation of PM-L and PM-F, 44 g of the ^137^Cs-intact-litter or the decomposed-litter-mixture were added to 2.6 kg of PM, respectively. Plastic containers (400 mm wide × 300 mm deep × 145 mm high) were filled with each of the potting mixes.

Eight komatsuna plants were grown in each potting mix (PM, PM-L and PM-F). The containers were kept in a greenhouse under natural illumination for 30 days (growing period). The temperature was controlled at 25 ± 5 °C during the growing period. Planting was independently repeated three times at the same period.

The edible parts of komatsuna were harvested on day 30 after planting, and they were dried at 80 °C in a drying oven. After the harvest, the potting mixes were air dried, and then three subsamples were collected from each dried potting mix. The dried plants and soil samples were powdered with a grinder (Labo Milser LM-PLUS, Osaka Chemical Co., Ltd.), and each powdered sample was put into a U8 container for the analysis of ^137^Cs.

### Measurement of ^137^Cs

The activity concentrations of ^137^Cs in the samples were determined by a gamma spectrometry method using a high-purity germanium detector (GMX-type, ORTEC, Seiko EG&G, Tokyo, Japan). The detector was calibrated with volume radioactivity standard gamma sources (MX033U8PP, Japan Radioisotope Association). The standard reference material JSAC-0471 (the Japan Society for Analytical Chemistry) was used for an accuracy check. The activity concentrations were corrected for radioactive decay to the sample collection day.

### Soil-to-plant transfer factors

The soil-to-plant transfer factors (TFs) were calculated as the ratio of the activity concentration of ^137^Cs in the plant (Bq/kg-dry weight) to its concentration in the potting mix (Bq/kg-dry weight).

### Statistical analysis

Significant differences in the amount of harvested komatsuna were determined by one-way analysis of variance (ANOVA) with Tukey’s HSD post hoc test. Student’s *t*-test was carried out to confirm the effect of decomposition on the extraction of ^137^Cs from leaf litter.
